# Gay, Bisexual, and Other Men Who Have Sex with Men’s Experiences of Intimate Partner Violence in Four Celtic Nations: A Mixed-Method Study

**DOI:** 10.1177/08862605251331077

**Published:** 2025-04-12

**Authors:** Steven Maxwell, Kareena McAloney, Dimitra Strongylou, Rosaleen O’Brien, Rosie Stenhouse, Jamie Frankis

**Affiliations:** 1 Glasgow Caledonian University, UK; 2 Independent Research Consultant, Glasgow, UK; 3 University of Edinburgh, UK

**Keywords:** gay and bisexual men, intimate partner violence, domestic abuse, sexual violence

## Abstract

This study investigates the prevalence, forms, and unique cultural manifestations of intimate partner violence (IPV) among gay, bisexual, and other men who have sex with men (GBMSM) in four Celtic nations (Scotland, Ireland, Northern Ireland, and Wales) while recognizing differences due to gendered heteronormativity. The research involved a cross-sectional survey of 1,389 participants and in-depth narrative interviews with 10 individuals, with recruitment conducted via GBMSM social and sociosexual media platforms. The study employed descriptive statistics to estimate IPV prevalence and types, while logistic regression was used to identify socio-behavioral predictors of IPV. Findings suggest that the 1-year IPV prevalence among GBMSM was 33% (*n* = 546), with emotional abuse (13%) and belittlement (13%) as the most common forms. Notably, all qualitative participants reported experiencing coercive control. The narratives indicated that IPV predominantly occurred in longer-term relationships, with no instances identified in singular intimate encounters. The study found that while the prevalence of IPV among GBMSM is similar to that observed among heterosexual women, the nature of IPV and the recognition of certain forms of nonconsensual sex as IPV differ between these populations. These differences can be better understood through the lens of masculinities discourses and the heteronormative framework of Western societies. The study underscores the importance of building a dedicated body of evidence on IPV within sexual minority communities to inform policy and provide appropriate support through health and social care initiatives. Addressing IPV in GBMSM relationships is crucial for safeguarding this community and contributing to the broader societal goal of eradicating violence.

## Introduction

The World Health Organization (WHO, 2012) defines intimate partner violence (IPV) as acts by a former or current intimate partner that involve physical, sexual, or psychological violence, and/or controlling behaviors that negatively impact the person’s health. Mainstream narratives present IPV as perpetrated by men against women, but evidence suggests it may be as common in male intimate relationships (Rollè et al., 2018; WHO, 2021). Prevalence studies from North America (Bacchus et al., 2017; Ducan et al., 2018; Stults et al., 2015) and the United Kingdom (Milz et al., 2019) estimate that 34% to 45% of men in same-sex relationships ever experience IPV. Gay, bisexual, and other men who have sex with men (GBMSM) who experience IPV from male partners have increased risks of mental illness, substance abuse and sexually transmitted infections (Bacchus et al., 2017; Ducan et al., 2018; Milz et al., 2019; Stults et al., 2015, 2019). Evidence from North America found that social discourses of masculinity and gay stereotypes, such as the perception that IPV only affects heterosexual women, assumptions that men should be able to defend themselves, and stereotypes of effeminacy and equal power dynamics in same-sex male relationships can prevent GBMSM from self-identifying as victims of IPV and consequently from accessing services (Baker et al., 2013; Ristock & Timbang, 2005). In addition, when GBMSM do access services for support, their experiences are often not recognized as IPV, partly due to heteronormative biases in support systems shaped by wider social norms (Rohrbaugh, 2006), with some also experiencing discrimination within these systems (Rollè et al., 2018). Thus, GBMSM may experience iatrogenic harm from engagement with services.

Heteronormativity refers to societal structures, practices, and norms privileging heterosexual relationships, often marginalizing non-heterosexual ones and rendering minority experiences invisible (Robinson, 2016). Studies examining GBMSM IPV experiences are predominantly undertaken using standardized definitions and measures based on a heteronormative perspective, limiting the ability to examine relationship dynamics unique to GBMSM (Baker et al., 2013). For example, standardized IPV assessment tools are validated for heteronormative power imbalances, such as male-perpetrator and female-victim dynamics, which are not reflective of male-male intimate relationship dynamics. Similarly, there are IPV activities that are specific to GBMSM (e.g., stealth-breeding, nonconsensual condom removal, and ejaculation during anal sex between men; Brennan, 2017), while bondage, discipline, dominance and submission (BDSM) (which may include consensual physically violent behaviors which are therefore not IPV) is more prevalent among GBMSM communities (Sprott, 2023).

While most GBMSM IPV research is single method and/or originates from North America, there are limited studies from the Celtic nations: Scotland, Wales, Northern Ireland, and the Republic of Ireland. By integrating mixed-method approaches through a non-heteronormative lens, this study aims to address these gaps and provide a deeper understanding of GBMSM IPV within the cultural contexts of these regions. Our study objectives were (a) To estimate the prevalence of IPV within a sample of GBMSM in four Celtic nations; (b) to understand socio-behavioral characteristics related to IPV; (c) to understand how GBMSM recognize and define their experiences as IPV; and (d) to understand how IPV experiences manifest in the context of GBMSMs relationships.

## Methods

### Design

This study employed a multi-method design, combining a quantitative cross-sectional survey with loosely structured narrative interviews, recruiting GBMSM primarily online across four Celtic nations. Ethical approval was obtained from the joint first authors’ University Ethics Committee (HLS/NCH/19/019 and HLS/NCH/21/004).

### Quantitative Recruitment

The social media, men who have sex with men, sexual and holistic health (SMMASH3) study is the third iteration of a serial, cross-sectional online survey conducted within four Celtic nations; Scotland, Wales, Northern Ireland, and the Republic of Ireland (e.g., Frankis et al., 2016; McGarty et al., 2021; Strongylou et al., 2022; Whiteley et al., 2023). The questionnaire (see https://www.smmash2020.org/surveys) was developed by an interdisciplinary steering group that included community members and administered via REDCap, an online survey application. Strongylou and Frankis (2021) provide a detailed account of the development and administration of SMMASH3. In summary, GBMSM using gay-facing geo-sociosexual networking websites and apps were targeted with message “blasts” and banner advertisements promoting and linking to the survey. Additionally, paid advertising was used on Facebook to target men who “liked” a range of LGBTQIA+ related social issues, and local commercial gay venues and third-sector organizations also posted survey invitations on Twitter (now X). Inclusion criteria were GBMSM (allowing participants to self-define so in practice this included cismen, transmen, nonbinary folk, and a small number of participants who identified as female/transwomen) wishing to, or having sex with, men; and aged ≥16 if located in Scotland, Wales, and Northern Ireland, or ≥17 if located in the Republic of Ireland, based on the local age of sexual consent. Participants who clicked on advertisements were taken to an information sheet explaining the voluntary nature of the study and confidentiality of any data collected, before linking to the survey itself. Recruitment took place between December 2019 and mid-March 2020, just before the national COVID-19 lockdowns.

### Quantitative Measures

The survey measured sociodemographic, behavioral, and mental health (see Table 1) and IPV (see Table 2) experiences. Recent depression and anxiety were measured with the Patient Health Questionnaire 9 (PHQ-9; Kroenke et al., 2001) and Generalized Anxiety Disorder-7 (GAD-7; Spitzer et al., 2006) questionnaires, which assess depression and anxiety severity, respectively, over the past 2 weeks. PHQ-9 scores range from 0 to 27, with 0 to 9 indicating none/mild depression and 10 to 27 indicating moderate/severe depression (Levis et al., 2019). GAD-7 scores range from 0 to 21, with 0 to 9 indicating none/mild anxiety and 10 to 21 indicating moderate/severe anxiety (Manea et al., 2015). Though neither are diagnostic tools, individuals with moderate/severe scores would typically be considered for clinical intervention (Manea et al., 2015). Hazardous drinking in the last year was measured using the Fast Alcohol Screening Test (see Hodgson et al., 2002), which is considered a valid tool (Meneses-Gaya et al., 2010). Questions assessing IPV experiences were derived from pre-existing scales but explicitly modified for GBMSM with additional items developed specifically for this study. Five items were taken from the *Sex and Relationships Problems* scale (Bailey et al., 2013; Table 2, items #1–5), reworded for GBMSM (e.g., “without your consent” was added to item #3 to acknowledge GBMSMs higher prevalence of BDSM sex; De Neef et al., 2019). A further eight questions (Table 2, #6–13) were developed and tested by our steering group to measure additional aspects of IPV that GBMSM may experience, drawing on current literature and recent cultural phenomena (e.g., “stealth breeding” and nonconsensual sharing of naked photos). We asked participants about their experiences of IPV in the last year, rather than the narrower 3-month period used by Bailey et al (2013), to capture recent, if not ongoing, experiences, in line with our other behavioral questions. Because of the extremely sensitive nature of IPV, participants were initially asked whether they were prepared to view these questions and provided with local IPV support services and resources onscreen; those who declined were routed to the next section of the survey.

**Table 1.** Demographics and Health Behaviors of the Survey Sample.

	Whole Sample (*N* = 1,389)		No IPV^a^ (*n* = 876)	63.1%	Reported IPV^a^ (*n* = 513)	36.9%
	*n*	%	*n*	%	*n*	%
Country						
Scotland	687	49.5	429	62.4	258	37.6
Wales	190	13.7	125	65.8	65	34.2
Northern Ireland	105	7.6	65	61.9	40	38.1
Republic of Ireland	407	29.3	257	63.1	150	36.9
Age						
16–25	198	14.3	117	59.1	81	40.9
26–35	374	26.9	219	58.6	155	41.4
36–45	347	25.0	212	61.1	135	38.9
46+	470	33.8	328	69.8	142	30.2
Gender						
Male	1,357	97.7	866	63.8	491	36.2
Transgender/nonbinary/female	32	2.3	10	31.3	22	68.8
Sexual orientation						
Gay	1,152	82.9	729	63.3	423	36.7
Bisexual/straight/other	237	17.1	147	62.0	90	38.0
Ethnicity						
White	1,345	96.8	853	63.4	492	36.6
People of color or mixed ethnicity	44	3.2	23	52.3	21	47.7
Relationship status						
Single	799	57.5	491	61.5	308	38.5
Regular male partner^b^	489	35.2	319	65.2	170	34.8
Regular female partner^b^	101	7.3	66	65.3	35	34.7
HIV status						
HIV+	106	7.6	65	61.3	41	38.7
HIV−/unsure	1,283	92.4	811	63.2	472	36.8
HIV/STI test in the last year						
No	443	31.9	319	72.0	124	28.0
Yes	946	68.1	557	58.9	389	41.1
Financial worries						
Occasionally/never	825	59.4	560	67.9	265	32.1
Sometimes/all of the time	564	40.6	316	56.0	248	44.0
Gay scene attendance						
At least 2–3 times a month	294	21.2	148	50.3	146	49.7
Once a month or never	1,095	78.8	728	66.5	367	33.5
Sex party in the last year						
No	915	65.9	612	66.9	303	33.1
Yes	474	34.1	264	55.7	210	44.3
Chemsex^c^ in the last year						
None	1,260	90.7	817	64.8	443	35.2
At least some Chemsex	129	9.3	59	45.7	70	54.3
Anxiety score (GAD-7)						
None/mild	1,101	79.3	757	68.8	344	31.2
Moderate/severe	288	20.7	119	41.3	169	58.7
Depression score (PHQ-9)						
None/mild	971	69.9	690	71.1	281	28.9
Moderate/severe	418	30.1	186	44.5	232	55.5
Illicit drugs (including cannabis) in the last 4 weeks						
No	1,103	79.4	719	65.2	384	34.8
Yes	286	20.6	157	54.9	129	45.1
Fast alcohol screening tool						
Safe	908	65.4	599	66.0	309	34.0
Hazardous	481	34.6	277	57.6	204	42.4
Smoking/vaping						
Current smoker/vaper	379	27.3	216	57.0	163	43.0
Nonsmoker or ex-smoker	1,010	72.7	660	65.3	350	34.7

*Note.* GHB = gamma-hydroxybutyrate; GBL = gamma-butyrolactone; IPV = intimate partner violence; PHQ-9 = Patient Health Questionnaire 9; GAD-7 = Generalized Anxiety Disorder-7; STI = sexually transitted infection.

a For these columns, proportions are reported *across* rows.

b Including civil partnerships and marriage.

c Chemsex is the use of specific drugs (usually mephedrone, crystal methamphetamine, ketamine, GHB, and/or GBL) to enhance sexual situations; see Frankis et al. (2018).

**Table 2.** Proportion of Participants Reporting Each of our 13 IPV Items.

Item	In the Last Year, Have You (Been). . .	*N*	%
1	Humiliated or emotionally abused in other ways by a partner or ex-partner?		
	No	1,195	86.0
	Yes	180	13.0
	PNS	11	1.0
2	Afraid of a partner or ex-partner?		
	No	1,260	90.7
	Yes	121	8.7
	PNS	8	0.6
3	Forced to have any kind of sexual activity by a partner or ex-partner?		
	No	1,289	92.8
	Yes	84	6.0
	PNS	16	1.0
4	Kicked, hit, slapped, or otherwise physically hurt by a partner or ex-partner without your consent?		
	No	1,272	91.6
	Yes	109	7.8
	PNS	8	0.6
5	Told by a partner who you could see and where you could go?		
	No	1,261	90.8
	Yes	116	8.4
	PNS	12	0.8
6	Put down or told you are worthless by a partner or ex-partner?		
	No	1,209	87.0
	Yes	173	12.5
	PNS	7	0.5
7	Forced to use drugs before or during sex?		
	No	1,348	97.1
	Yes	31	2.2
	PNS	10	0.7
8	Kissed or touched against your will?		
	No	1,220	87.8
	Yes	158	11.4
	PNS	11	0.8
9	Experienced “stealthing” or “stealth-breeding” (having a partner remove a condom without your agreement)?		
	No	1,207	86.9
	Yes	70	5.0
	PNS	7	0.5
	I’ve never heard of this	105	7.6
10	Forced to have any kind of sexual contact?		
	No	1,293	93.1
	Yes	87	6.3
	PNS	9	0.7
11	Filmed/photographed during sex against your will?		
	No	1,280	92.2
	Yes	92	6.6
	PNS	17	1.2
12	Has someone shared/posted a naked photo of you without your agreement?		
	No	1,245	89.6
	Yes	129	9.3
	PNS	15	1.0
13	Passed out during sex or can’t remember what happened during sex?		
	No	1,255	90.4
	Yes	128	9.3
	PNS	6	0.4

*Note.* IPV = intimate partner violence; PNS = prefer not to say or missing.

### Quantitative Data Analysis

Quantitative data were screened to exclude participants who were not in the four Celtic nations during data collection, those who did not answer our IPV questions, or had missing data on any of the regression variables. Participants who answered yes to any of the 13 IPV questions were deemed to have experienced IPV in the last year. SPSS V29 (IBM) was used to calculate descriptive, univariate inferential, and multivariate regression analyses. The lack of evidence predicting IPV experiences for GBMSM meant we chose an exploratory statistical approach (backward binary logistic regression) to examine this issue. Estimated odds ratios (ORs) and 95% confidence intervals were calculated to explore factors associated with reporting IPV in the past year among GBMSM across the four nations. The final model contained all variables significant at the bivariate level (*p* < .05) to assess which remained statistically significant.

### Qualitative Recruitment

A digital poster with study information was placed on multiple Scottish GBMSM charities’ social media accounts alongside three advertisements on GBMSM geo-sociosexual networking apps (Recon, Scruff, and Grindr) between June and July 2022. Potential participants contacted the research team via email and were screened for eligibility as follows: they were aged 18 years and over, resided in Scotland, identified as GBMSM, self-identified as having experienced abuse from an intimate male partner, believed they would not be at risk of harm from perpetrators by taking part in the study, and felt safe and secure in their well-being to discuss their IPV experiences. Qualitative recruitment was focused on Scotland to streamline logistics, engage with local communities more effectively, and allow for a deeper, contextually relevant exploration of IPV. This focus complemented the broader quantitative method across the Celtic nations, ensuring both depth and national scope. We wanted to explore how participants conceptualized IPV from a non-heteronormative viewpoint, so no formal definition was used and participants self-identified as experiencing IPV.

Participants in the qualitative study received written participant information and had the opportunity to ask the research team questions during the recruitment process prior to providing consent for arranging an interview. Consent was reaffirmed and recorded at the interview. A practitioner from a GBMSM health charity was available to discuss any concerns men had about participating. Participants could withdraw from the study at any time without providing a reason. Support was available to participants if needed following the interview. Pseudonyms were applied and other potential identifying information was removed from transcripts to ensure anonymity. All data was managed in accordance with data protection laws and university data management protocols.

### Individual Narrative Interviews

Loosely structured narrative interviews were conducted in July 2022 via Zoom or telephone, designed to elicit participants’ stories of IPV. This narrative approach allowed stories to be told from within participants’ frames of reference, structured in a way that made sense to them (Stenhouse, 2013). Each interview began with an invitation for participants to tell their stories, followed by three broad, open-ended questions. The interview design prioritized participant autonomy and well-being, allowing them to set boundaries around topics they wished to avoid, safeguarding against over-disclosure (Stenhouse, 2013). Interview questions were informed by a priori framework, shaped by the study’s objectives and existing literature. This framework covered types of abuse, health impacts of IPV, and interwoven relationship dynamics while allowing flexibility to explore both predefined and emergent themes. To ensure confidentiality, participants were asked to conduct the interviews in private spaces. Interviews averaged 75 minutes, were audio-recorded, and transcribed verbatim by a professional transcription service.

### Qualitative Data Analysis

Data analysis followed a two-phase process. Initially, a holistic narrative focus was applied to individual interviews to identify overarching patterns and key elements (Lieblich et al., 1998). In the second phase, a thematic analysis of the entire dataset integrated the initial a priori framework and emergent themes arising directly from participants’ narratives. This iterative process ensured that the analysis remained grounded in participants’ lived experiences while being aligned with the study’s objectives. The thematic analysis followed Terry et al.’s (2017) five-stage process: (a) Familiarization with the data, (b) development of a coding framework incorporating both a priori and emergent themes, (c) coding of all data, (d) thematic mapping, to explore patterns, contradictions, and silences across the dataset, and (e) writing and refining an interpretative narrative. The initial loose a priori framework was adapted throughout the analysis as emergent themes enriched and challenged the original structure. For example, coercion and control emerged as a dominant theme spanning various types of IPV, shaping relationships across the dataset. This iterative and flexible approach allowed for a nuanced understanding of the complexities of GBMSM IPV. The analysis was conducted by one researcher, with the coding framework reviewed by two other authors at the early and final stages to ensure reliability and mitigate bias. MAXQDA 22 was used for data management.

## Results

### Survey Participant Characteristics

A total of 1,856 participants completed the survey, of whom 7.2% (*n* = 163) declined to see the IPV questions. A further 304 had missing data on the regression variables, meaning *n* = 1,389 participants were included in this analysis. Their socio-behavioral characteristics are shown in Table 1. The majority (*n* = 687, 49.5%) were from Scotland, and one-third were aged ≥46 years with younger people (16–25, *n* = 198, 14.3%) underrepresented. Nearly all identified as male (*n* = 1,357, 97.7%) with 2.3% identifying as trans/nonbinary/female, and while most identified as gay (*n* = 1,152, 82.9%), a substantial minority reported bisexual, heterosexual, or other identities (*n* = 237, 17.1%). Most were white (*n* = 1,345, 96.8%) and while over half (*n* = 799, 57.5%) were single, one-third reported a regular male partner (including civil partnerships and marriage, *n* = 489, 35.2%). Overall, 7.6% (*n* = 106) had tested HIV positive, and over two-thirds (*n* = 946, 68.1%) reported a sexually transmitted infection (STI) or HIV test in the last year. Two-fifths (*n* = 564, 40.6%) reported financial worries “some or all of the time.” Most (*n* = 1,095, 78.8%) attended the local commercial gay scene once a month or less on average. While one-third (*n* = 474, 34.1%) reported attending a sex party in the last year, just 1 in 10 (*n* = 129, 9.3%) reported chemsex (as defined in Table 1: C) in the same period. One-fifth (*n* = 228, 20.7%) reported moderate-to-severe anxiety, and almost one-third (*n* = 418, 30.1%) moderate-to-severe depression. Over one-third (34.6%, *n* = 481) reported hazardous drinking, and just over one-quarter (27.3%, *n* = 379) were current smokers or vapers.

### Interview Participant Characteristics

Ten participants who met the inclusion criteria completed an interview. All the participants identified as gay or bisexual and their ages ranged from 26 to 47 years (median 32 years; interquartile range 27–42). Most had experienced IPV from one male partner within a long-term relationship, lasting between 2 and 13 years. However, a few participants had experienced IPV within shorter, seemingly intense relationships, lasting a maximum of only a few months. All the narratives were of relationships that had ended a year or more prior to the interview.

### IPV Prevalence and Types of Abuse

A substantial minority of survey participants (*n* = 163, 7.2%) declined to see the IPV questions, suggesting our prevalence findings are conservative estimates. Over 1 in 3 (36.9%, *n* = 513) reported that they had experienced IPV in the previous year (Table 1). Table 2 summarizes participant responses for the 13 IPV items. Emotional abuse (#1, 13%) and being put down (#6, 12.5%) were the most frequently reported, followed by being kissed or touched against your will (#8, 11.4%), having naked photos shared without consent (#12, 9.3%), passing out or being unable to remember what happened during sex (#13, 9.3%), being afraid of a partner (#2 8.7%), being told who you could see or where you could go (#5, 8.4%) and experiencing nonconsensual physical violence (#4, 7.8%) from a partner/ex-partner. Being filmed or photographed during sex against your will (#11, 6.6%), forced to have any kind of sexual contact (#10, 6.3%) or sexual activity with a partner/ex-partner (#3, 6.0%), and experiencing “stealth breeding” (#9, 5.0%) were reported reasonably often (although notably, an additional 7.6% of participants had never heard of stealth breeding before) but being forced to use drugs before or during sex (#7, 2.2%) was comparatively rare.

### Bivariate Analyses

We examined the socio-behavioral differences between those who did (*n* = 513, 36.9%) and did not (*n* = 876; 63.1%) report IPV in the last year. Chi-squared (χ^2^) analysis suggested that participants who were under 46 years old (χ^2^ = 14.30, *p* < .005), trans/nonbinary/female (χ^2^ = 14.24, *p* < .001), used the gay scene at least twice a month (χ^2^ = 25.93, *p* < .001), reported financial worries (χ^2^ = 20.2, *p* < .0001), an HIV or STI test (χ^2^ = 22.33, *p* < .001), sex party participation (χ^2^ = 16.78, *p* < .001), chemsex (χ^2^ = 18.34, *p* < .001), illicit drug use (χ^2^ = 10.33, *p* < .001), hazardous drinking (χ^2^ = 9.48, *p* < .005) or smoking/vaping (χ^2^ = 8.26, *p* < .005) in the last year, or moderate/severe anxiety (χ^2^(1,1) = 73.76, *p* < .001) and depression (χ^2^ = 88.52, *p* < .001) in the last 2 weeks were all significantly more likely to report IPV in the last year. Study country, sexual orientation, ethnicity, relationship status, and HIV status were not related to reporting IPV.

### Logistic Regression Analysis

Table 3 presents the results of the backward binary logistic regression analysis predicting reporting IPV in the last year. Variables significant at the bivariate level were entered into the constant model. Table 3 shows the predictive independent variables included in the final model, which was set in four steps. This means that the odds of reporting IPV were; 3.3 times more likely for transgender/nonbinary/female people than cisgender men; 1.3 times more likely for those who reported experiencing financial worries most or all of the time, 1.7 times more likely for those who attended the gay scene frequently (at least twice a month) compared to those who did not (once a month or less); 1.6 times more likely for those who report an HIV/STI test in the last year; 1.4 times more likely for those who report sex party attendance in the last year; 1.8 times more likely for those who report moderate/severe anxiety; and 2.2 times more likely for those who report moderate/severe depression than those who do not. None of the remaining variables were significant predictors of reporting IPV in the final model. The internal validity of the model was good (Hosmer-Lemeshow = 5.1, *p* > .05, confirming goodness of fit).

**Table 3.** Constant Only and Final Logistic Regression Model—Coefficients of the Model Predicting Reporting IPV in the Last Year.

Predictors	*B*	SE	95% CI for Odds Ratio			
			Sig.	Lower	Odds	Upper
Constant model						
Gender (male)	1.187**	0.413	0.004	1.459	3.277	7.360
Financial worries	0.268*	0.123	0.029	1.027	1.307	1.663
Gay scene frequency	0.559***	0.146	<0.001	1.314	1.749	2.327
HIV/STI test	0.440**	0.137	0.001	2.033	1.553	1.186
Sex party	0.304*	0.134	0.023	1.044	1.356	1.762
Chemsex	0.346	0.207	Ns	0.942	1.414	2.124
Anxiety	0.562**	0.177	0.001	1.240	1.754	2.482
Depression	0.790***	0.158	<0.001	1.617	2.204	3.005
Hazardous drinking	0.208	0.126	Ns	0.963	1.232	1.576

*Note*. SE = standard error; IPV = intimate partner violence; CI = confidence interval; STI = sexuall transmitted infection.

* *p* < .05. **p < .01. ***p < .001.

### Qualitative Findings

The findings are presented within a framework that reflects the iterative nature of narrative inquiry, structured around five key themes: (a) Vulnerabilities: Factors increasing susceptibility to IPV, (b) recognizing: Processes of identifying abusive behavior, (c) trajectories: Patterns of IPV escalation and progression, (d) coercion and control: Central dynamic in relationships, intersecting key IPV types, and (e) abuse dynamics: GBMSM-specific manifestations of financial, sexual and physical IPV. This structure underscores the interwoven, underscoring the interconnected and multifaceted nature of traumatic experiences, while ensuring authenticity and grounding in participants’ narratives.

### Vulnerabilities to IPV

Several participants suggested characteristics that they thought might increase their vulnerability to IPV such as autism spectrum disorder, or geographical isolation from their social networks. These factors created opportunities for manipulation and control by abusive partners. A few participants noted that abusive partners exhibited vulnerabilities, such as past experiences of abuse or childhood trauma, which led them to position the abuser as the “vulnerable victim” (Will, 30–40 years), thereby excusing or rationalizing IPV behaviors.

### Recognizing IPV

Most participants only recognized the behaviors as IPV with insight from friends or professionals, or with hindsight following the termination of the relationship.

I wouldn’t have been able to say, ‘this is intimate partner violence’. I wouldn’t have been able to label it. . ..my friend. . . probably knew what was going on . . .from the outside it’s obvious. But it wasn’t obvious to me at all. (Ryan, 30–40 years)

Some participants shared how they had ignored early warnings, rationalizing or minimizing the partner’s abusive acts as “insecurities” (David, 40–50 years). Others, despite having doubts about what was happening in the relationship, identified that avoiding loneliness and maintaining long-sought intimacy were influencers for remaining with an abusive partner.

I didn’t know anybody down here, so he was my only sort of friend . . .even though I knew it was wrong, I still just kept on going back and letting it happen. (Blair, 30–40 years)

Some participants limited understanding and experience of intimate relationships made it difficult to distinguish IPV behaviors from what might be considered the “ups and downs of the relationship” (Lucas, 20–30 years), leading them to minimize or dismiss abusive acts.

### IPV Trajectories

All participants reported experiencing multiple abuse types identified in the survey, including physical, sexual, financial, psychological, and coercion and control. Initial experiences, such as put-downs or other verbal abuse, often escalated over time, becoming more frequent and increasingly violent both psychologically and physically.

It started with looks and snide comments. It was, ‘Oh don’t wear that, that makes you look fat’, and other comments like that. I just thought that was the way all partners were and because it was often passed off as a joke. So, that continued, probably for a good three months and then it started becoming more and more aggressive” (Grant, 30–40 years).

Participants described abuse types as interwoven, with coercion and control emerging as central within narratives, driving the trajectory of escalating IPV and shaping relationships.

### Coercion and Control

Coercion and control emerged as a central theme across all the participant narratives, shaping and serving as mechanisms for enacting various types of abuse.

The predominant early form of coercion and control was psychological abuse, particularly “gaslighting”, where perpetrators’ behaviors made participants constantly question their own reality. Other behaviors included what Lucas (20–30) described as “emotional blackmail and guilt-tripping to force me to do something I didn’t want or as punishment for wrongdoing.”

Social forms of abuse were also an early common means of exerting control, including constant surveillance of text messages/phone calls, locking participants in or out of their homes, and confiscating personal items like phones and keys.

He’d call me all the time. He *was* very insistent on messaging me, making sure where I was and who I was talking to and things. He would. . . expect me to call him every day at ten o’clock. . . If I was late, I’d get a shit storm. I always had to like to do what he wanted when he wanted. (Matt, 20–30 years).

Reflecting on their experiences, participants identified control as the unifying thread across all types of IPV. On participant noted:

There is a similar common theme through it all, all types of IPV, it’s just the possession, the controlling. . . When your partner becomes controlling and possessive, [whether] it’s through intentional physical harm, through sex and intimacy, and just through everyday communication. (Lucas, 20–30 years).

Ultimately, participants perceived control not only as a tactic but as the primary goal of IPV, regardless of the specific forms of abuse their partners employed. The following section explores financial, sexual, and physical forms of abuse, which were often intertwined with psychological and social IPV, serving as early mechanisms for coercion and control.

### Financial Abuse

For most participants, financial abuse was intertwined with broader patterns of control, often manifesting as exploitation when they had greater financial resources or accommodation. This included partners relying on them for money, taking control of bank accounts, demanding the purchase of alcohol or drugs, or moving into their homes without consent.

He didn’t work, and I’ve got a good job and get paid quite a lot. I didn’t mind helping him out, because obviously he was just on benefits. It then just sort of became bad. He was smoking quite a lot of cannabis. . .that would usually be the sort of tipping point, where he was struggling to get money and then he would just really kick off. (Blair, 30–40 years).

Some participants highlighted challenges in extricating themselves when their abusive partners gained control of their living arrangements.

He just kind of moved into my flat without me even really agreeing to it. Then, before I knew it, all his stuff was here. (Ollie, 30–40 years).

It got bad when we were living together, and I decided I wanted to break up with him, but I couldn’t go anywhere because that’s where I lived. Basically, I had paid all the deposit and all the bills. . . I was quite strapped. . . It was thousands of pounds that he owed me. He gave me it back in bits and pieces for months and months, just to, I don’t know, prolong things or something. (Ryan, 30–40 years)

These accounts illustrate financial abuse as a tactic of control, with abusive partners displaying entitlement to participants’ resources and leaving some participants to manage debts incurred in their name long after the relationship ended.

### Sexual Violence, Coercion, and Consent

The theme of control extended into the realm of sexual violence; with several participants describing being verbally coerced into having sex with their partners. Within these narratives, consent was often ambiguous; participants reflected that, while they had seemingly agreed to sex, they did so despite feeling unwilling.

I would just let him do it. . . get it over with. (Will, 30–40 years)

One participant described that he just went along with his partner’s demands, to the extent that he was no longer able to determine if he was consenting or not.

I never had sex with him if I didn’t want to, but he would sometimes put pressure on me. He would never keep having sex with me if I said I wanted to stop. . .but I don’t know if I would have known if I didn’t want to or not sometimes. (Ryan, 30–40)

Several participants recounted experiences of being forced into various sexual acts against their will, some of whom did not recognize their experiences as rape. These participants’ accounts included unwanted anal penetration or forced oral sex. Participants also shared difficulties in negotiating sexual boundaries, including condom use and other protective measures. For example, partners insisted on condomless anal sex (without or before HIV preexposure prophylaxis was available), refused regular HIV and STI testing, or coerced participants into sex with multiple partners. A few participants experienced nonconsensual condom removal (*stealth breeding*), during anal sex, which caused distress and was exacerbated by a lack of relevant information specific to male-male relationships.

I would insist on using a condom. We were having sex, and he took the condom off and then he continued. I hadn’t realised until afterwards. . .I remember Googling ‘my partner takes the condom when having sex, what do I do?’ and it was all women that were replying. There were no gay instances of it. (Matt, 20–30 years)

These narratives underscore the layered complexities of consent, coercion, and boundary negotiation in abusive relationships, compounded by the absence of tailored information. As coercion and control intensify, they often create conditions in which physical violence emerges.

### Physical Violence

Most participants described physical violence as developing later in relationships, ranging from several weeks to a few years. They detailed the first act of violence, noted the escalation of violence over time, and identified coercive and controlling acts as precursors. Physical assaults sometimes occurred in public, where bystanders would observe and not intervene. Many participants minimized the violence they experienced, referring to it as “just smacking” or “not extreme.” Some felt ashamed for not stopping smaller or younger partners from harming them and worried that their appearance, particularly perceptions of strength or masculinity, might lead others to doubt their victimization. A few participants sought to mitigate the threat of violence by building up their bodies at the gym, aiming to counter perceived vulnerability and enhance their sense of protection.

I allowed it and I thought, well, he’s young, he’s a lot smaller than me. A lot of the times I could hold him back, but it just got so bad, I ended up in hospital. (Blair, 30–40 years).

### Triangulation of Results

Our mixed-methods approach allows us to triangulate our data, presenting a more nuanced understanding of the complex relational context in which participants experienced IPV. Our survey data suggest that around one-third of GBMSM in our sample experienced aspects of IPV within the last year, using our inclusive definition (rather than men defining their experience as IPV), with qualitative data exploring the relationship context within which it took place. While multiple types of abusive behavior were quantified in the survey, interview data describe how this manifests, clarifying that men do not always recognize these experiences as abusive, and the complex narratives around control, violence, loneliness, and consent that GBMSM navigate. Finally, while our regression analysis highlights the links between IPV and gender identity, financial worries, sociosexual behaviors, and mental health, our interview data uncover unique barriers to service access and reporting IPV to the police due to men’s sexual minority status.

## Discussion

This mixed-methods study, the largest to examine IPV experienced by men in same-sex relationships recruited from an online sample, extends our understanding of the prevalence and scope of IPV. We measured IPV outside of a heteronormative definition, adding items specific to GBMSMs sociosexual cultures. Moreover, in exploring men’s IPV narratives we found they do not readily identify their experiences as abusive and lacked clarity about sexual consent, while barriers unique to men’s sexual minority status prevented them from leaving their abusive partner and impacted police reporting and service access. Finally, we identified multiple predictors of IPV which have impacts on policy and service development.

We found that one-third (34.6%) of GBMSM in the Celtic nations experienced IPV in the last year. As 7.2% of participants declined to see these questions, this may be considered an underestimate. This estimate of annual prevalence is higher than other U.K. and North American literature, which found 34% to 45% of GBMSM will ever experience IPV (Bacchus et al., 2017; Callan et al., 2021; Ducan et al., 2018; Milz et al., 2019) in their *lifetime* and suggests that the rate of IPV in intimate male relationships in the Global North is similar to women in heterosexual relationships (Rollè et al., 2018). While our more inclusive measure may underpin this higher prevalence, including items relevant to GBMSM sociosexual culture (e.g., “stealth-breeding” (Brennan, 2017); acknowledging that *wanted* violence within a consensual BDSM scene is not IPV (Sprott, 2023); etc.) mean we have used a more culturally nuanced measure.

All 13 IPV items were reported across our survey participants and disclosed within our qualitative interviews. In line with other studies (Bacchus et al., 2017; Milz et al., 2019; Rollè et al., 2018), emotional abuse and nonconsensual sex were the most frequently reported items, while fear, controlling behaviors, unwanted physical violence, exploitative video/photography, and economic abuse were relatively commonplace. Within the qualitative data, a complex trajectory of IPV behaviors was described as escalating over time, from gaslighting as emotional abuse to increasingly violent episodes both physically and psychologically. However, participants often failed to recognize experiences as abuse which for some was influenced by relationship inexperience or were reluctant to acknowledge it due to fear of loneliness and losing an intimate connection, leading them to stay with their partners.

Our study highlighted ambiguity in determining consensual and nonconsensual sex, shaped by heterosexual social norms that hindered men from identifying abusive sexual acts. This resonates with the work of Finneran and Stephenson (2014) and Javaid (2018) who found that societal norms concerning masculinity and sexual consent may contribute to the absence of rape narratives among men who have experienced sexual violence. Societal expectations that men should defend themselves against unwanted sexual advances were key to our participants’ narratives and hindered GBMSM from acknowledging and reporting instances of sexual abuse (Goldenberg et al., 2016; Finneran & Stephenson, 2014), leading to under-recognition and underreporting. Indeed, our participants described how characteristics related to hegemonic masculinity and dominance, including age, size, and muscularity, were incongruous with identifying themselves as “victims.” Moreover, the absence of gender-based power expectations in same-sex relationships can lead to conflicts arising from psychosocial inequalities, where one partner ultimately serves to attain hegemonic masculinity by exerting control over the other’s decision-making and displaying emotional strength (Finneran & Stephenson, 2014). Clearly, a critical redefinition of masculinity, gender norms, and consensual sex narratives is urgently required.

Several factors were significant predictors of IPV. Transgender women and nonbinary people were over three times more likely to report IPV than cisgender men (OR = 3.2), somewhat higher than previous studies have found (Peitzmeier et al., 2020). This increase may result from the increasingly toxic climate transgender women face in the Global North, and, coupled with the multiple wider health inequalities they experience (Reisner et al., 2016), calls for greater service provision and, critically, inclusion for these gender minority people. Men with financial worries were more likely to report IPV (OR = 1.3), which resonates with other studies of IPV among same-sex couples (Rollè et al., 2018). However, while financial coercion and control was a common experience for most qualitative participants, it was the financially secure partner who was typically exploited. This adds a crucial dimension to our understanding of financial abuse among GBMSM and demonstrates a clear departure from the hegemonic heterosexual IPV narrative. Finally, poor mental health was, perhaps unsurprisingly, related to IPV with those experiencing moderate-to-severe anxiety (OR = 1.8) or depression (OR = 2.2) significantly more likely to report IPV. While this has been well documented elsewhere (Milz et al., 2019; Rollè et al., 2018) this is likely a bidirectional relationship, with poor mental health making men more vulnerable to exploitative relationships (Milz et al., 2019), and the result of such exploitation.

Overall, unique IPV factors related to masculinity and social norms distinctly threaten GBMSM victims’ health, demanding more inclusive IPV policies and practices.

## Limitations

One notable strength of this study was the deliberate avoidance of predefined IPV definitions and the implementation of culturally adapted measures, enabling a nuanced understanding of IPV from a non-heteronormative perspective. This approach allows for the inclusion of diverse relationship dynamics and experiences that are often overlooked in traditional, heteronormative frameworks. However, a limitation inherent in this methodology is the potential bias introduced by basing any measures on constructs originally derived from heterosexual dynamics and prevailing societal norms. Consequently, there may be an inadvertent reinforcement of these norms, which could obscure the unique aspects of IPV in non-heteronormative relationships and limit the generalisability of the findings. Despite this, the key strength of providing a more inclusive and representative exploration of IPV outweighs the limitations commonly found in other studies that rely on rigid, heteronormative definitions and measures.

Alongside the generic criticisms of one-off, cross-sectional, online surveys, we mainly recruited through gay sociosexual media, which are largely used to seek new sexual partners. Thus, we likely gathered a relatively sexually active sample and potentially missed men who are currently in abusive relationships (where surveillance and controlling behaviors may make sociosexual media use difficult). We also under-recruited younger men (<26 years), which is of some concern since IPV was related to younger age in the univariate analyses, and our four study countries were also unequally represented. However, while financial and technology literacy barriers to access such media also exist, previous studies have highlighted the relative similarity of such online samples to other GBMSM recruitment methods (e.g., via gay commercial venues) (e.g., Frankis et al., 2018; https://doi.org/10.1071/SH17159), and it is currently impossible to recruit a probability sample of GBMSM in our target nations. Thus we argue that our data are likely reasonably generalizable to the wider population of GBMSM in these four Celtic nations. Recruiting interview participants only in Scotland as a purposive sample potentially limited the transferability of the qualitative findings. However, our mixed-methods study design strengthens rigor, addressing at least some of these limitations.

## Implications

The study has implications for our community’s understanding, identification, and reporting of IPV as well as support provisions. High levels of male IPV underscores its status as a public health concern that challenges heteronormative-based policy responses. However, the difficulties experienced by qualitative participants in recognizing IPV suggests many will not seek support, while the apathetic reception men reported from service providers suggests an urgent need for service development. We recommend drawing on the inclusive, nonjudgmental approach sexual health services have long adopted, as well as providing training for wider services to better recognize and support the unique IPV experiences of GBMSM.

## Conclusions

This study presents the largest mixed-methods study to examine GBMSM IPV. Around one-third of the GBMSM sample experienced IPV, which manifests in multiple, complex forms, predominantly emotional and controlling behaviors. However, GBMSM participants found it difficult to identify these experiences as IPV, leading to a lack of reporting. Coupled with a lack of intimate male IPV understanding among the police and health providers, our work highlights the need for equitable, inclusive IPV policies and services that effectively address the unique needs of GBMSM and underscores the need for further research with trans/nonbinary people.
